# Microbial effects of cold-pressed Sacha inchi oil supplementation in rats

**DOI:** 10.1371/journal.pone.0319066

**Published:** 2025-02-20

**Authors:** Tepparit Samrit, Supawadee Osotprasit, Athit Chaiwichien, Phawiya Suksomboon, Supanan Chansap, Thitikul Suthisintong, Narin Changklungmoa, Pornanan Kueakhai

**Affiliations:** Food Bioactive Compounds Research Unit and Faculty of Allied Health Sciences, Burapha University, Chonburi, Thailand; Wuhan Polytechnic University, CHINA

## Abstract

Oil supplements have various benefits for metabolism, particularly Sacha inchi oil (SI), which is rich in polyunsaturated fatty acids (PUFAs) such as ω-3 and fat-soluble vitamins. However, the impacts of oil supplements on gut health remain unclear. The aim of this study was to compare the effects of an SI supplement with those of lard oil (LO), known for its high saturated fatty acid content, and a normal diet on gut health in male Sprague Dawley rats for 12 consecutive weeks. Fecal DNA was used to assess gut microbiota diversity and species abundance, diversity, and function prediction. Colon tissue from each rat was examined for colon crypt depth and histology. Rats administered the LO supplement exhibited higher dysbiosis than those administered the SI supplement, with the LO supplement influencing the relative abundance of various bacteria at the genus level. A KEGG analysis was conducted to examine the effects on metabolic pathways, revealing that the SI supplement promoted carbohydrate metabolism while reducing immune system activity. In contrast, the LO supplement increased replication, repair, and translation activities. A histological analysis of the colon tissues showed no significant alterations in crypt depth or lesions in all groups, indicating that neither supplement induced adverse structural changes in the gut. The results of this study suggest that SI supplementation modulates the gut microbiota, thereby enhancing gut health and metabolic function.

## Introduction

Sacha inchi (*Plukenetia volubilis*), which is commonly found in tropical regions, is notable for its high omega-3 content [1]. Previous reports have found that it provides benefits for many diseases. Studies on rats have shown that SI supplementation can improve metabolic syndrome induced by a high-fat diet through improving metabolic status [2] and the gut microbiota [3]. The dosage of oil supplements in previous studies was about 15 ml (or one tablespoon) for humans. The metabolic rate of rats is approximately 10-fold higher than that of humans. When comparing doses, one tablespoon (15 ml) in a 60 kg human corresponds to a ratio of 0.25 ml/kg, which is equivalent to 2.5 ml/kg in a rat [4–6]. SIO is extracted using the cold press method and is highly valued commercially. Additionally, there is high demand for SIO in worldwide markets, where it is commonly utilized as a dietary supplement. SIO has a high content of essential fatty acids, such as α-linolenic acid (ALA, ω-3) [7]. However, previous studies have reported that dietary fats at normal doses can affect gut health, including the gut microbiota and gut histology [8].

The gut is an important part of the body and possesses many functions for the digestion and absorption of dietary macro- and micro-nutrients. The gut microbiota consists of microorganisms living in the gastrointestinal tract, with approximately 10^13^ to 10^14^ microbes and more than 1000 species of bacteria, the majority of which are anaerobic bacteria [9]. The gut microbiota is related to health status, as it can modulate inflammatory and anti-inflammatory activities by producing short-chain fatty acids (SCFAs) in the intestine, which affect G-protein receptors on target cells [10]. This is associated with many diseases, such as central nervous system disorders [11], chronic gastrointestinal diseases [10,12], and metabolic syndrome [13,14]. The factors that regulate the gut microbiota include environmental and dietary factors. However, the impacts of dietary components, such as fiber and fatty acids, on the gut microbiome are highly significant in terms of altering its diversity [14].

Dietary lipids are important macronutrients that contain an abundance of fatty acids. Fatty acids are used for energy expenditure and as a source of fat-soluble vitamins. Recent studies have found that oil is not only used for energy expenditure and as a source of fat-soluble vitamins, but also provides benefits for gut health by modulating the gut microbiota [15,16]. Three types of fatty acids have been identified in oil: SFAs, monounsaturated fatty acids (MUFAs), and polyunsaturated fatty acids (PUFAs) [16,17]. SFAs have been found in cheese, butter, palm oil, coconut oil, and lard oil (LO). A high SFA intake is associated with increased intestinal permeability, insulin resistance [14,18], and impaired gut barrier function, as it increases lactobacillus and firmicutes but decreases Bacteroidetes and Bifidobacteria [19,20]. In contrast, some oils are abundant in PUFAs, such as ω-3 and ω-6. In a previous study, it was found that ω-3 can restore and improve the gut microbiota and gut health by activating an anti-inflammatory response [21–23]. SFAs cause inflammation in the intestine, which can protect it from harmful infections. However, excess SFAs can induce excessive inflammation, leading to intestinal damage. Therefore, to maintain a balance between inflammation and anti-inflammation, it is important to consume ω-3 and ω-6 fatty acids together in an appropriate ratio [24,25]. Therefore, this study aimed to investigate the effects of SIO on the gut microbiota and gut histology, specifically to assess its impact on gut health at a normal dose. The effects were compared with those in a control group receiving distilled water and another group receiving LO.

## Materials and methods

### Oil extraction and preparation

Sacha inchi (PBM-005750) brown fruits were obtained from TAI.C.M.S. Standard Industrial Co., Ltd., Mueang Chiang Rai, Thailand. They were cultivated in Chiang Rai, Thailand, for 5-6 months. The extraction process involved peeling the Sacha inchi brown fruits (3 months) and drying them at 120 °C for 10 min. After that, the fruits were pressed to extract SIO [26] and filtered to remove impurities. LO is a rendered lard oil that was purchased from a supermarket and produced in Thailand (lot number 010621). All oils were stored at 4 °C before administration.

### Animals and experimental design

Male Sprague Dawley rats (7 weeks old) from Nomura Siam International Co. Ltd., Thailand, were randomly divided into three groups (7 rats/group): normal diet (ND), SI, and LO groups. All rats were given standard rodent chow, and filtered water was available ad libitum. The rats were maintained under control conditions, with a constant room temperature of 22 ±  1 °C, a relative humidity of 30–70%, and a 12/12-hour (h) light/dark cycle. The oils were stored at 4 °C before being aliquoted and warmed to room temperature using a water bath prior to administration. The ND group (control group) was treated using distilled water. The rats were administered the treatments via oral gavage at 2.5 ml/kg body weight every day for 12 weeks. At the end of the animal experiment, the rats were fasted for 16 h and then euthanized with an excess dose of thiopental sodium and thoracotomy. After euthanasia, all rats underwent necropsy, and their colons and feces were collected. The colons were washed with phosphate-buffered saline and fixed with 4% paraformaldehyde [26]. The feces were collected from the rectum. The feces from the ND (n = 6), SI (n = 4), and LO (n = 5) groups were stored at −80 °C for a gut microbiota analysis. This study was approved by the Biosafety Board of Burapha University (IBC 038/2564, approval date: 25 January 2022), the Institutional Animal Care and Use Committee (IACUC 008/2564, approval date: 14 May 2021), and the Animal Care and Use Committee Thammasat University (012/2021, approval date: 20 September 2021).

### DNA extraction from fecal

DNA from the gut microbiota of each rat was extracted from 100 mg of feces using a ZymoBIOMICS DNA Miniprep Kit (California, USA), according to the manufacturer’s protocols. The fecal samples from each rat were added to ZymoBIOMICS™ lysis solution and homogenized in ZR BashingBead™ lysis tubes for 40 minutes with vortexing. Subsequently, the samples were purified using a Zymo-Spin™ III-F filter and mixed with ZymoBIOMICS™ DNA binding buffer. The DNA in the samples was bound to a Zymo-Spin™ IICR column and washed with ZymoBIOMICS™ DNA wash buffer. In the final step, the samples were purified using a Zymo-Spin™ III-HRC filter and ZymoBIOMICS™ HRC Prep solution again and stored at −20 °C until analysis.

### PCR amplification of 16S rDNA

The DNA extracted was amplified using the polymerase chain reaction (PCR). The V3-V4 variable region of 16S rDNA of bacteria was amplified by forward and reverse PCR degenerate primers (341Forward: 5´-ACTCCTACGGGAGGCAGCAG-3´ and 806Reverse: 5´ GGACTACHVGGGTWTCTAAT-3´). PCR reaction was performed in a 50 μL reaction containing 30 ng template and primers. The PCR conditions started at 95 °C for 3 min, 30 cycles of 95 °C for 15 sec, 56 °C for 15 sec, 72 °C for 45 sec, and final extension at 72 °C for 5 min. The PCR products were then purified using DNA magnetic beads (BGI, LB00V60). Agilent 2100 Bioanalyzer was used to measure the concentration and size of the libraries.

The final double-stranded library products were denatured to generate single-stranded library products. The reaction was then performed to obtain single-strand circularized DNA products. Any remaining single-strand linear DNA was digested and removed. The single-strand circularized library was amplified using phi29 and rolling circle amplification (RCA) to generate DNA nano balls (DNBs), which contain multiple copies of the initial single-stranded library molecule. The DNBs were loaded onto the patterned nanoarray, and sequencing reads of PE300/250 base lengths were generated using the DNBSEQ-G400 platform.

### Sequence analysis from DNA libraries

The raw sequencing data were filtered to generate high-quality, clean data. Next, clean reads that shared common regions were merged into tags and subsequently grouped into operational taxonomic units (OTUs). OTU representative sequences were taxonomically classified using the Ribosomal Database Project database. A sequencing data analysis was performed using USEARCH (v7.0.1090) software. Sequences in paired-end reads were merged using FLASH [27]. USEARCH (v7.0.1090) was used to organize the tags into Operational Taxonomic Units (OTUs). The tags were grouped into OTUs based on a 97% similarity threshold using UPARSE, with unique sequences used to identify each OTU. Chimeras, which are sequences mistakenly assembled from two or more different original sequences, were identified and removed using UCHIME (v4.2.40). For specific types of sequences such as 16S rDNA and ITS, chimeras within the OTUs were identified and removed by comparing them against the gold standard database (v20110519) and the UNITE database (v20140703), respectively. Finally, all tags were matched to their corresponding OTU representative sequences using USEARCH GLOBAL [28].

Further analysis included OTU Venn maps, alpha diversity, beta diversity, the relative abundance of microbial species, different microbiota structures, and a differential functional analysis. KEGG (Kyoto Encyclopedia of Genes and Genomes) levels were used to show the differences between the gut microbiota groups and their functions. An OTU Venn map of the three groups was constructed and displayed using Venn Diagram of software R (v3.1.1). Alpha diversity indicated by species richness and diversity within samples was analyzed via observed species, Chao, Ace, Shannon, Simpson, and Good’s coverage using R software (version 3.2.1). Beta diversity indicated by species richness and diversity among samples was analyzed using QIIME (version 1.80), and the R software (version 3.4.1). The taxonomic breakdown of the OTU representative sequences was conducted using the Bayesian algorithm of the RDP classifier to determine the microbial composition in the R software (version 3.4.1). Species abundance was determined across seven taxonomic levels (phylum, class, order, family, genus, and species). The data on species abundance are displayed in a bar plot; species with a relative abundance below 0.5% were grouped into a category labeled “others”. The LefSe algorithm was employed for statistical significance testing to identify distinctive features and associated species classification (https://huttenhower.sph.harvard.edu/galaxy/). The LefSe results (LDA scores >2) are displayed in a cladogram. Microbial functional annotation was predicted using PICRUSt2 (Phylogenetic Investigation of Communities by Reconstruction of Unobserved States) in terms of KEGG level.

### Colonic histological analysis

Colon tissues from each rat were fixed in 4% paraformaldehyde for 24 h and then embedded in paraffin and dehydrated. This process involved sequential immersions in ethanol solutions of increasing concentrations: 70% ethanol for 1 h; 80% ethanol for 1.5 h; 90% ethanol for 1.5 h; 95% ethanol for 1.5 h; and, finally, absolute ethanol three times, each for 1 h. Subsequently, the tissues were treated with xylene three times, each for 1 h, followed by two 2 h immersions in paraffin and, finally, they were embedded in paraffin again. The tissues in the paraffin block were sectioned to a 5 µm thickness using a manual rotary microtome (Leica, Wetzlar, Germany) and transferred to silane-coated glass slides. After the sectioning process, the colon tissues were stained with hematoxylin and eosin (H&E). The staining procedure began with 5-minute (min) immersions in xylene, followed by 3-minute immersions in absolute ethanol. This was succeeded by dehydration phases using ethanol solutions of 95%, 80%, and 70% concentrations, applied sequentially for 3 min each, a sequence that was then repeated. After dehydration, the samples were rinsed in tap water for 5 min, subjected to 3 min staining with Harris hematoxylin (Bio-Optica, Milan, Italy), and then rinsed once more in tap water for 15 min or until the rinse water ran clear. In the next step in the rehydration process, ethanol solutions of increasing concentrations (70%, 80%, and 95% ethanol for 3 min each) were used. Following this, the samples were stained with alcoholic eosin (Bio-Optica, Milan, Italy) for 2 min, and they were dehydrated twice using 95% ethanol for 2 min each time and then twice more in absolute ethanol for 3 min each. In the final step, the tissues were cleared for 3 min in xylene 2 times and were mounted with Bio Mount™ (Bio-Optica, Milan, Italy), and images were captured (Olympus BX43 and DP23-AOU, Tokyo, Japan).

The morphology and pathology of the colon tissues were evaluated using a light microscope. Ten fields from each section were randomly selected for assessment, with two sections per group analyzed using the Naini and Cortina score for the histological activity score in inflammatory bowel disease [29,30]. Colonic crypt depths were measured using an ImageJ analyzer [31], and evaluations were made from ten crypt structures per image, across five images from each rat, with each group [32].

### Statistical analysis

Colonic crypt depths are expressed as means ±  SD and were analyzed using a one-way ANOVA. Differences between individual treatment groups were compared using Tukey’s test. Statistical significance in the gut microbiota was analyzed using the Wilcoxon rank-sum test, LDA, and size effect. In this research, differences between groups were considered significant at P <  0.05. The results and charts were analyzed and illustrated using GraphPad Prism (Graphism, Inc., USA, version 7), the R software (version 3.2.1 and 3.4.1), and QIIME (version 1.80).

## Results

### Effect of oil supplementation on gut microbiota diversity

**Fig 1:**
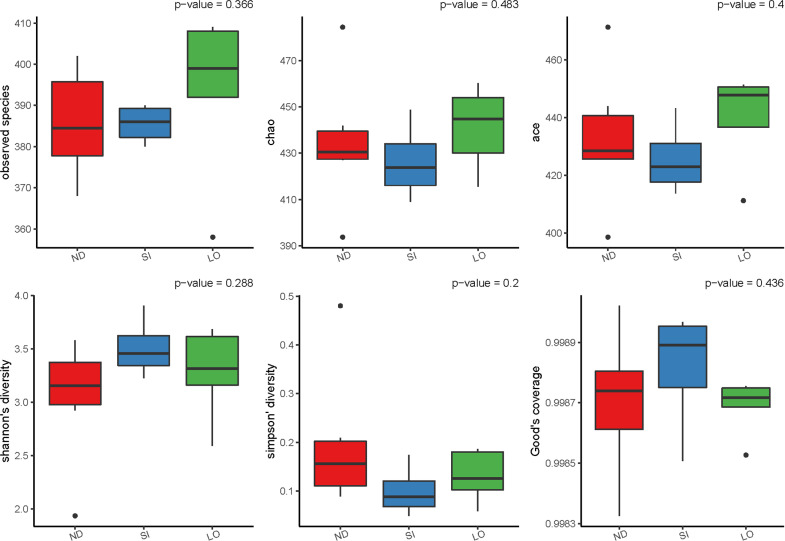
Each boxplot displays the distribution of alpha diversity, including observed species, Chao, ACE, Shannon’s diversity, Simpson’s diversity, and Good’s coverage.

**Fig 2:**
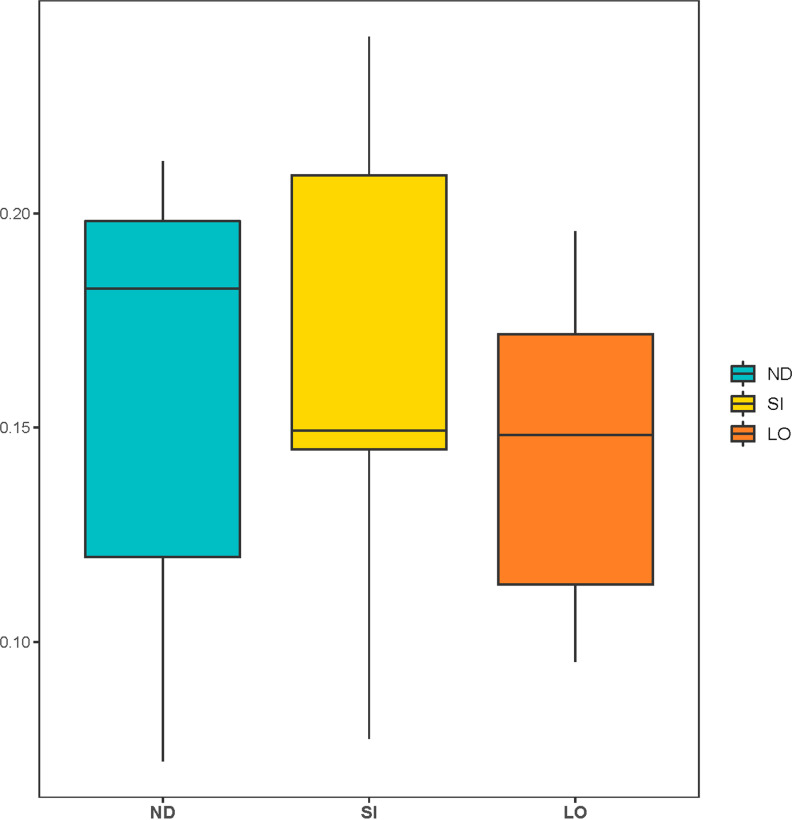
Boxplot presents the Bray-Curtis beta diversity between three groups: ND (turquoise), SI (yellow), and LO (orange) groups. Comparison of the distribution and variability of beta diversity measures among the groups.

The differences in the number and distribution of gut microbiota were analyzed; diversity is measured in terms of the similarity or dissimilarity between two communities (beta diversity) and the diversity within a single sample (alpha diversity). The alpha diversity boxplots present the diversity of the three groups (Fig 1), which did not significantly differentiate in the observed species (*P* = 0.366), Chao (*P* = 0.483), ACE (*P* = 0.400), Shannon’s diversity (*P* = 0.288), Simpson’s diversity (*P* = 0.200), or Good’s coverage (*P* = 0.436) indices. The beta diversity of all rats was calculated using the Bray–Curtis method (Fig 2). None of the groups showed abnormal values. The SI group had the highest median beta diversity, while the LO group had the lowest. This is in agreement with the variability observed, where the SI group exhibited the highest variability.

**Fig 3:**
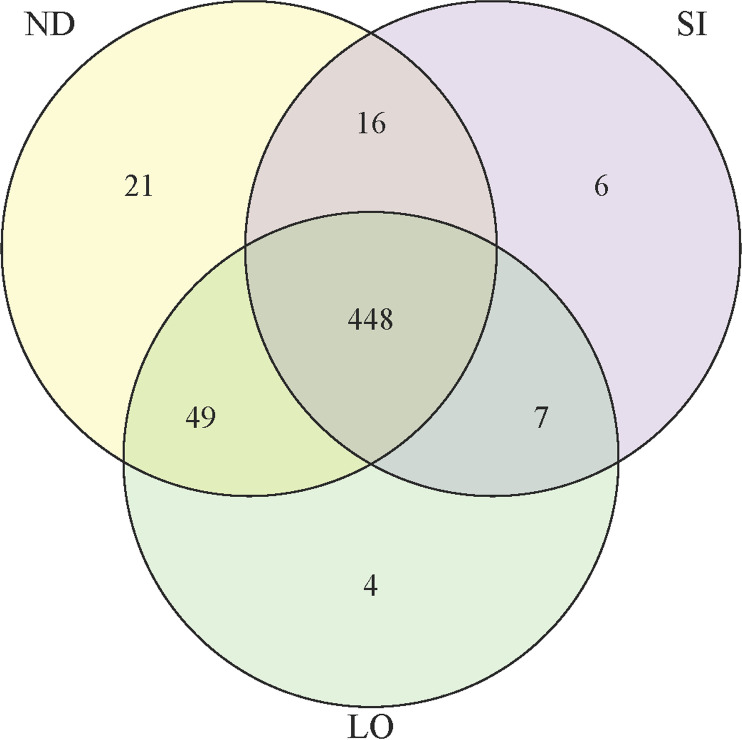
Venn diagram Illustrating shared OTUs among the ND, LO, and SI Groups. The yellow circle represents the ND group, the green circle represents the LO group, and the purple circle represents the SI group. The data in the overlapping areas represent the number of OTUs shared between groups, while the numbers in each non-overlapping area represent the number of OTUs unique to each group.

The diversity of the gut microbiota was analyzed, and a total of 563 OTUs were found across all groups. When comparing the number of samples sequenced and OTUs detected, it was found that the graph flattened out, indicating that the addition of more samples does not affect the increase in OTUs detected (S1 Fig). The OTU results are presented in a Venn diagram. Our results revealed a total of 551 OTUs. The ND group had a total of 534 OTUs, with 21 unique to ND and 65 shared with at least one other group. The SI group had a total of 477 OTUs, with 6 unique to SI and 471 shared with another group. The LO group had a total of 508 OTUs, with 4 unique to LO and 504 shared with another group. The ND group had the highest number of OTUs, and the ND and SI groups shared the greatest similarity, with 497 OTUs (Fig 3). An OTU Multiple Response Permutation Procedure (MRPP) analysis was used to compare the between- and within-group differences. The MRPP of the three groups was calculated using Bray–Curtis distances, with an A value of 0.0387, indicating that the difference between groups was greater than that within groups. The expected delta value was 0.4315 (*P* =  0.098), representing a greater difference between groups.

**Fig 4:**
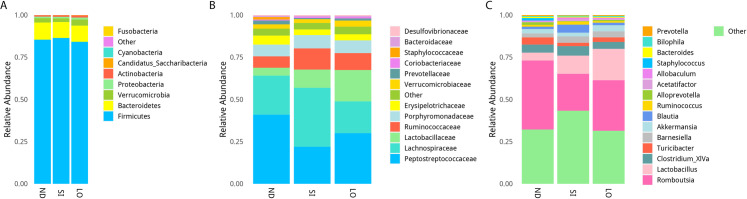
The effect of oil supplement on gut microbiota. The relative abundances of microbial species at the phylum (A), family (B), and genus levels (C).

**Fig 5:**
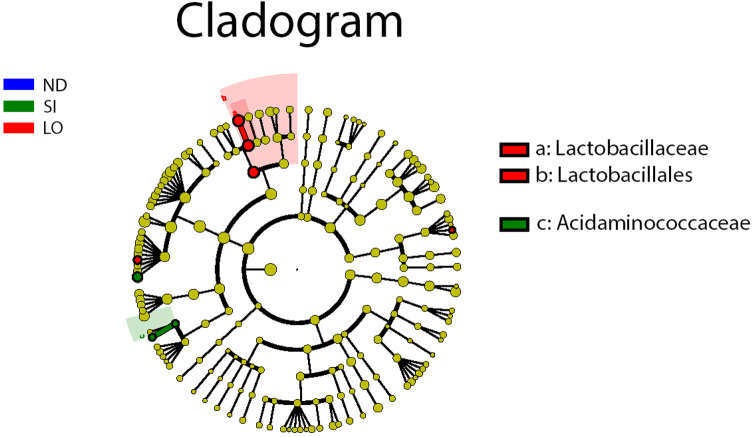
Different microbiota structures of ND, SI and LO groups detected by LEfSe tools (LDA scores > 2). The legends for biomarkers are displayed in the top-right corner. Yellow nodes signify biomarkers deemed unimportant across various groups. Progressing from the innermost to the outermost, the six concentric circles represent the taxonomic levels in the following order: phylum, class, order, family, genus, and species.

The groups did not show significant differences in relative abundance at the phylum level (Fig 4A). The SI and ND groups did not significantly differ at the family level; however, the LO group had significantly higher abundances of *Bifidobacteriaceae*, *Leuconostocaceae*, and *Veillonellaceae* and lower abundances of *Aerococcaceae* and *Prevotellaceae* than the SI group. When compared with the ND group, the LO group had higher abundances of *Bifidobacteriaceae* and *Lactobacillaceae* and lower abundances of *Acidaminococcaceae* and *Burkholderiaceae* (Figs 4B and S2). At the genus level, the SI group had significantly higher abundances of *Adlercreutzia* and *Anaerofustis* than the ND group, and it had higher abundances of *Aerococcus*, *Phascolarctobacterium*, and *Ruminococcus* and lower abundances of Bifidobacterium, *Collinsella*, *Faecalibacterium*, *Gemmiger*, *Megamonas*, and *Weissella* than the LO group. The LO group had significantly higher abundances of *Lactobacillus* and lower abundances of *Polynucleobacter* than the ND group. However, the LO group had slightly higher abundances of *Bifidobacterium*, *Collinsella*, *Faecalibacterium*, *Intestinimonas*, and *Lachnospiraceae* than the ND group (*P* = 0.052) (Figs 4C and S3). Furthermore, the differentiation of the gut microbiota using the Lefse algorithm demonstrated that the LO group began to show significant differences from the other groups at the family level, including in *Lactobacillaceae* (family level) and *Lactobacillales* (order level) compared to the ND and SI groups. Additionally, *Acidaminococcaceae* at the family level increased significantly in the SI group (Fig 5 and S1 Raw Data).

**Fig 6:**
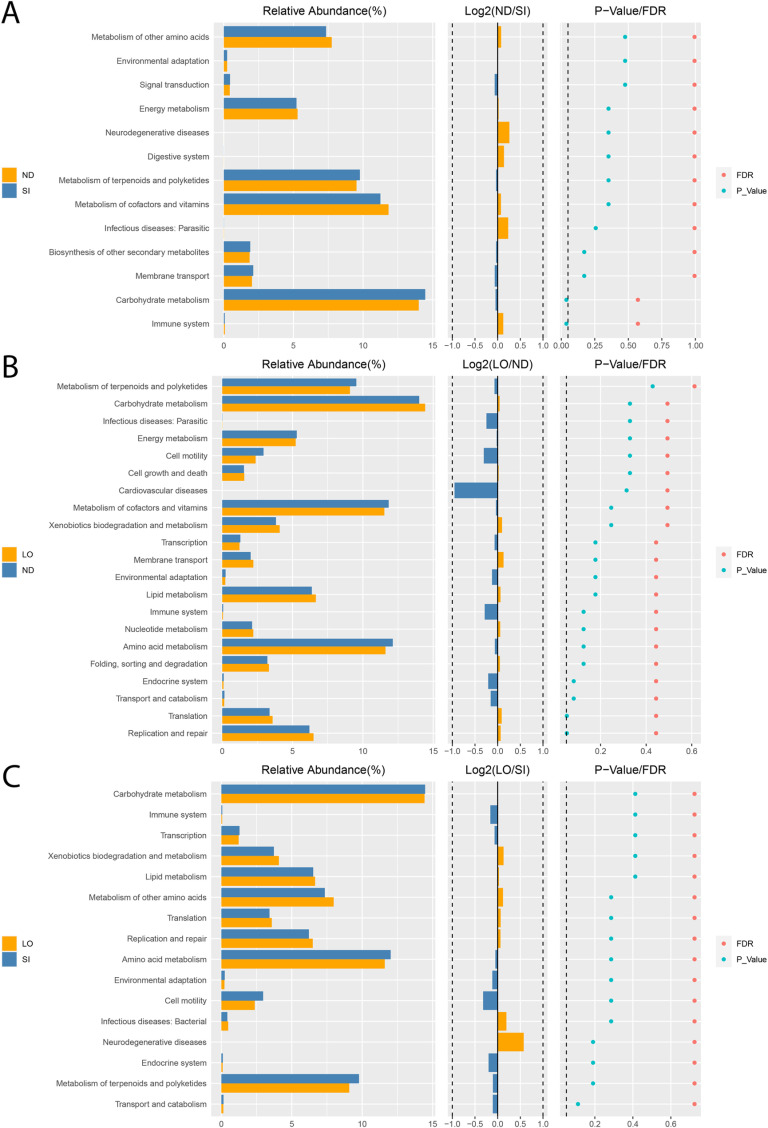
Differential function analysis levels 2 KEGG (Kyoto Encyclopedia of Genes and Genomes). Bar plot presenting the relative abundance of species. In the middle, the log2 values represent the ratio of the average relative abundance between two groups. On the right, P-values and FDR values derived from the Wilcoxon test are displayed. Comparisons are made between ND and SI (A), LO and ND (B), and SI and LO (C).

KEGG categories provide a means to classify genomes, biological pathways, and diseases with specific functional genes and predict their functions. A differential functional analysis revealed the predicted metabolic functions of the gut microbiota in feces (Fig 6). The groups showed no significant differences at KEGG level 1 (S4 Fig). The ND group had a higher relative abundance of the immune system than the SI group (*P* = 0.038, FDR = 0.571) and a lower relative abundance of carbohydrate metabolism than the SI group (*P* = 0.038, FDR = 0.571) at KEGG level 2. The KEGG level 2 analysis showed significant differences between the ND and LO groups in terms of replication, repair, and translation, with the LO group exhibiting higher levels than the ND group (*P* = 0.052, FDR = 0.444). The ND group presented a higher relative abundance of the immune system than the SI group (*P* = 0.038, FDR = 0.571) and a lower relative abundance of carbohydrate metabolism than the SI group (*P* = 0.038, FDR = 0.571). Between the ND and SI groups, six pathways were differentially abundant at KEGG level 3. The ND and LO groups had seven differentially abundant pathways. The LO and SI groups had significantly differentially abundant pathways in four areas (S5 Fig and S1 Raw Data).

### Effect of oil supplementation on colonic

**Fig 7:**
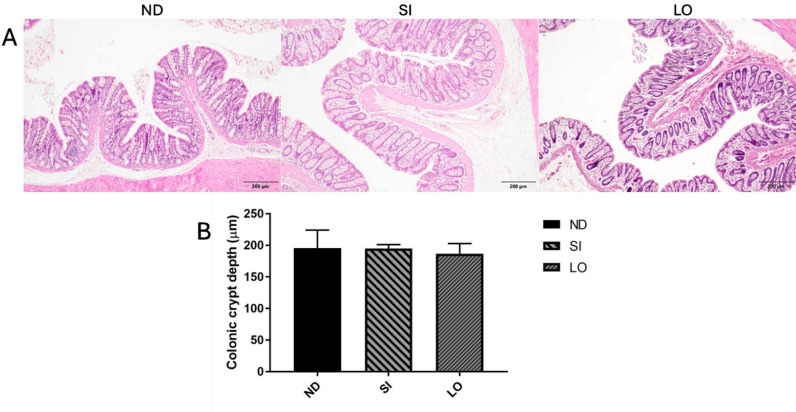
Effect of oil supplementation on colonic. The histology of the colon was analyzed using H&E staining and microscopy at 40 × magnification (A). The colonic crypt depth of each group was determined (B).

The histology of the colon was used to evaluate the functionality of the gut structure. An assessment using the Naini and Cortina score showed that none of the groups presented signs of inflammation, such as crypt architectural distortion, lamina propria eosinophils, granulomas, or lymph node hyperplasia (S1 Table). The results indicate that the rats given SI and LO did not exhibit changes in the structure or morphology of the colon when compared with the control group. This aligns with the observation that there was no significant difference in the colonic crypt depth between the groups (Fig 7).

In a previous study, all rats were given three types of oil, which did not change their body composition or blood chemical parameters such as liver enzymes and lipid profiles. Our results show that none of the oils altered the colon histology or colon length of the rats, but the rats given LO showed significant differences at the family level of the gut microbiota from the rats given distilled water (the ND group) and SIO (the SI group). According to our results, the major compounds of SI were omega-3 (LA: C18:3) at 44.73% and omega-6 (linoleic acid: C18:2) at 35.17% [26]. The results did not significantly differ at the phylum or family levels when compared with the control group. However, there was a significant difference at the family level, with a greater increase in *Aerococcaceae*, which is a member of the order *Lactobacillales*, phylum Firmicutes, and *Prevotellaceae*, a member of the order *Bacteroidales*, phylum *Bacteroidota*, than in the LO group. *Prevotellaceae*, known for being beneficial bacteria in the intestine, is correlated with vegetable consumption and is associated with improved cardiovascular disease risk factors and glucose metabolism [33–35]. In terms of glucose metabolism, *Prevotellaceae* is beneficial because it breaks down carbohydrates such as arabinoxylan from dietary polysaccharides and produces SCFAs [36,37]. *Prevotellaceae* has a high correlation with dietary fiber-induced improvements in glucose metabolism [38]. However, an overgrowth of *Prevotellaceae* can promote inflammation in the body and increase the risk of obesity and inflammatory bowel disease [39–41]. In our results, *Prevotellaceae* was abundant at baseline and did not exceed the levels found in the ND group. LO is an animal-source oil derived from leaf lard. The main fatty acid in LO is saturated fat. In our study, 15 ml of LO contained 14 g of total fat, including 4.5 g of saturated fat, 181.5 mg of ω-3, 2482.5 mg of ω-6, and 5124.0 mg of ω-9 (data obtained from the nutrition facts label). The gut microbiota in the LO group was different from that in the other groups. The LO group demonstrated an increased abundance of gut microbiota when compared to the ND and SI groups. Previous studies have reported that long-chain SFAs, particularly stearic acid, influence bile acid production and absorption, leading to abnormal lipid metabolism. This is due to the rat’s limited ability to absorb long-chain SFAs in the intestine. Fatty acids can influence the diversity and abundance of butyrate-producing bacteria in the gut microbiota. A low SFA intake results in a higher α-diversity than a high SFA intake, indicating that increased SFA consumption leads to decreased gut microbiota diversity and a lower abundance of butyrate-producing bacteria in humans. In contrast, polyunsaturated fatty acids (PUFAs) effectively improve lipid metabolism but have a lesser impact on gut microbiota than SFAs [42–44].

Omega-3 fatty acids are types of polyunsaturated fatty acids (PUFAs), including eicosapentaenoic acid (EPA), docosahexaenoic acid (DHA), and linolenic acid (LA). EPA and DHA are primarily found in animal-source oils, especially in fish oil, while LA is predominantly found in vegetable oil, with a high concentration in flaxseed oil [45,46] and SI [26,46–48]. LA is converted into EPA and DHA by enzymes. In humans, patients diagnosed with type 2 diabetes and given omega-3 from fish (DHA and EPA) showed a decrease in the *Firmicutes*/*Bacteroidetes* phyla ratio and an increase in the *Prevotella* genus [49]. In mice, fish oil significantly enriched the *Proteobacteria* phylum and reduced the abundances of *Roseburia* and *Phascolarctobacterium* at the genus level when compared with rats given lard and soy oil [45,50]. Regarding vegetable omega-3 (LA) from flaxseed, mice given flaxseed oil did not significantly differ in taxonomy from those on a base diet [50]. A previous study reported that a high-fat diet in rats given SI improved gut microbiota dysbiosis by protectively decreasing the relative abundance of *Firmicutes* and increasing the relative abundance of *Bacteroidetes*. Additionally, SI increased unidentified *Enterobacteriaceae*, *Bacteroides*, and *Lachnoclostridium* at the phylum level in rats given a high-fat diet [3].

A KEGG function analysis was carried out to analyze the metabolism of the rats in each group. At KEGG level 2, fish oil contains omega-3 fatty acids in the form of DHA and EPA. Rats given fish oil supplements had lower abundances of predicted changes in amino acid metabolism and enzyme families and higher abundances of predicted lipid metabolism and genetic information than the control group [8]. This is different from the rats given SI supplements, who had lower abundances of predicted immune system activity and higher abundances of predicted carbohydrate metabolism. The microbiota in the intestine affects the neutrophil and T-cell populations in the GI tract, which has beneficial effects on host defense and detrimental effects on activating inflammation [51]. High carbohydrate metabolism in the gut microbiota is beneficial for conditions associated with a high-fat diet and, compared to fat uptake through the gut microbiota, can be a potential therapeutic approach [52]. By comparing the ND and LO groups, it was found that the LO group exhibited a higher relative abundance of replication, repair, and translation than the ND group, correlating with bacterial growth [53,54].

Oil supplements have benefits for health status and help to protect against metabolic syndromes such as nonalcoholic fatty liver and diabetes in both humans and rodents [3,55]. However, consuming an imbalanced diet can induce abnormal functioning of the intestine, leading to diseases such as inflammatory bowel disease and ulcerative colitis. The digestive system is crucial for digestion and absorption, and it serves as a source of gut microorganisms that can produce short-chain fatty acids (SCFAs) and control the host’s metabolism, hence playing a significant role in health through gut histology [56]. Many studies have reported that rats fed a high-fat diet have colonic damage and a reduced colon crypt depth [57–59]. When supplemented with a high-fat diet, vegetable oils such as SI and olive oil can reduce the harm caused by a high-fat diet [3,60]. In addition, oil intake has been shown to cause abnormalities in the histology of the intestine. Saturated fats, such as palmitic and stearic acids, have pathological effects on the intestinal epithelium and cause inflammation [61]. It has been reported that, in Sprague Dawley rats, supplements with high levels of SFAs such as palm oil and LO affect colon histology by decreasing the colon crypt depth and inducing colonic ulcers when compared with other vegetable oils, including rapeseed, sunflower, and linseed oils, and control groups. This effect results from the low expression of mRNA for the Muc2 gene and increased IL-6 expression in the LO and palm oil groups [62–64]. However, in our results, the groups did not exhibit significant differences in colonic histology changes or colon crypt depth. These differences between previous studies and our study may be due to the age of the rats. The previous study used rats aged 4 weeks, while our study used rats aged 8 weeks. All results show that the rats given SIO and LO did not exhibit changes in the gut microbiota or colon histology when compared to the ND group. The LO group did show differences in the abundance of gut microbiota when compared to the ND group. However, when compared with the ND group, LO had a larger impact on changes in the gut microbiota than SIO. This initial finding could be further explored by increasing the sample size, prolonging the administration duration, testing specific fatty acids, or conducting studies in humans to understand the long-term effects of SIO supplementation.

## Conclusion

Our study found no significant differences in relative abundance at the phylum level across all groups. The SI supplement exerted a low influence on the type of gut microbiota compared to the ND group, while the LO supplement had a significant impact. At the genus level, the LO group exhibited not only higher levels of several beneficial bacteria, but also higher dysbiosis than the SI group. In a KEGG secondary metabolic pathway analysis, the LO supplement led to an increased abundance of replication, repair, and translation, whereas the SI supplement boosted carbohydrate metabolism and decreased immune system activity. This suggests that the SI supplement may activate carbohydrate metabolism in the intestine through bacteria, thereby reducing fat absorption. In a histological analysis of the colon, the rats showed no significant differences in colonic crypt depth and did not exhibit lesions or scores when evaluated using the Naini and Cortina score. From all the information presented, it can be concluded that the SI supplement has less adverse effects on gut health than the LO supplement, and can induce carbohydrate metabolism.

## Supporting information

S1 Fig Species Accumulation analysis. This graph presents the increase in species detected with the addition of each sample. The end of the graph starts to flatten out, indicating that increasing the number of samples does not significantly increase the number of OTUs detected. This suggests that the number of samples used in this analysis is sufficient for assessing species diversity.
(TIF)

S2 Fig The relative abundance of microbial species at the family level compared using the Wilcoxon test. The difference in microbial relative abundance between the ND and SI groups (A), the ND and LO groups (B), and the SI and LO groups (C).
(TIF)

S3 Fig The relative abundance of microbial species at the genus level compared using the Wilcoxon test. The difference in microbial relative abundance between the ND and SI groups (A), the ND and LO groups (B), and the SI and LO groups (C).
(TIF)

S4 Fig Differential function analysis levels 1 KEGG (Kyoto Encyclopedia of Genes and Genomes). The bar plot presents the relative abundance of species. In the middle, log2 values represent the ratio of the average relative abundance between two groups. On the right, *P*-values and FDR values derived from the Wilcoxon test are displayed. Comparisons are made between ND and SI (A), LO and ND (B), and SI and LO (C).
(TIF)

S5 Fig Differential function analysis levels 3 KEGG (Kyoto Encyclopedia of Genes and Genomes). The bar plot presents the relative abundance of species. In the middle, log2 values represent the ratio of the average relative abundance between two groups. On the right, *P*-values and FDR values derived from the Wilcoxon test are displayed. Comparisons are made between ND and SI (A), LO and ND (B), and SI and LO (C).
(TIF)

S1 Table The Naini and Cortina score of colonics in ND, SI and LO groups. (DOCX)

S1 Raw Data Raw data for relative abundance of microbial species and differential function analysis of KEGG (ZIP). (RAR)
