# Supplementary figures and images for: Microbial effects of cold-pressed Sacha inchi oil supplementation in rats
Source: PLoS One. 2025 Feb 20;20(2):e0319066. doi: 10.1371/journal.pone.0319066 (PMC11841868; doi:10.1371/journal.pone.0319066)

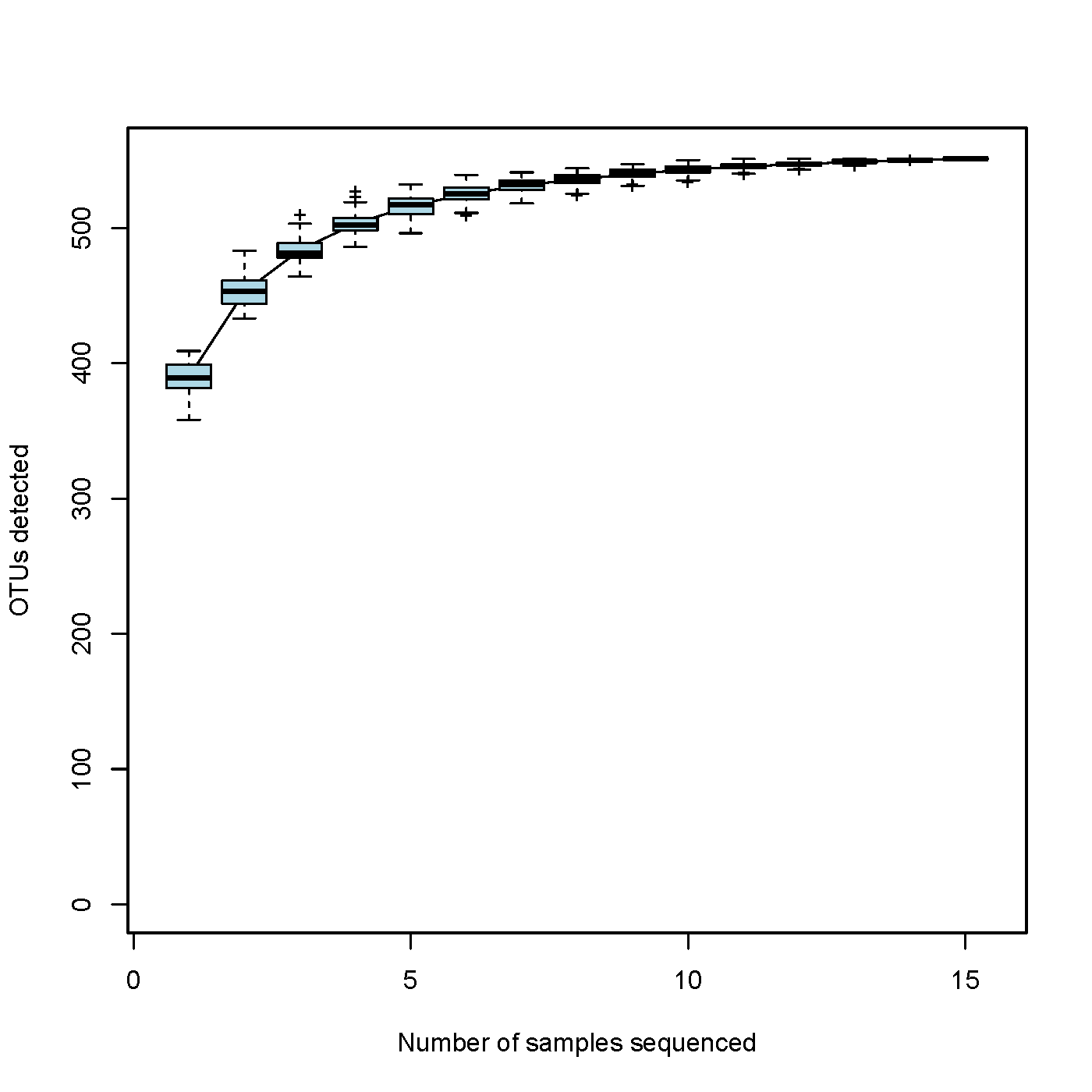

Supplement: S1 Fig — This graph presents the increase in species detected with the addition of each sample. The end of the graph starts to flatten out, indicating that increasing the number of samples does not significantly increase the number of OTUs detected. This suggests that the number of samples used in this analysis is sufficient for assessing species diversity. (TIF) [file pone.0319066.s001.tif]

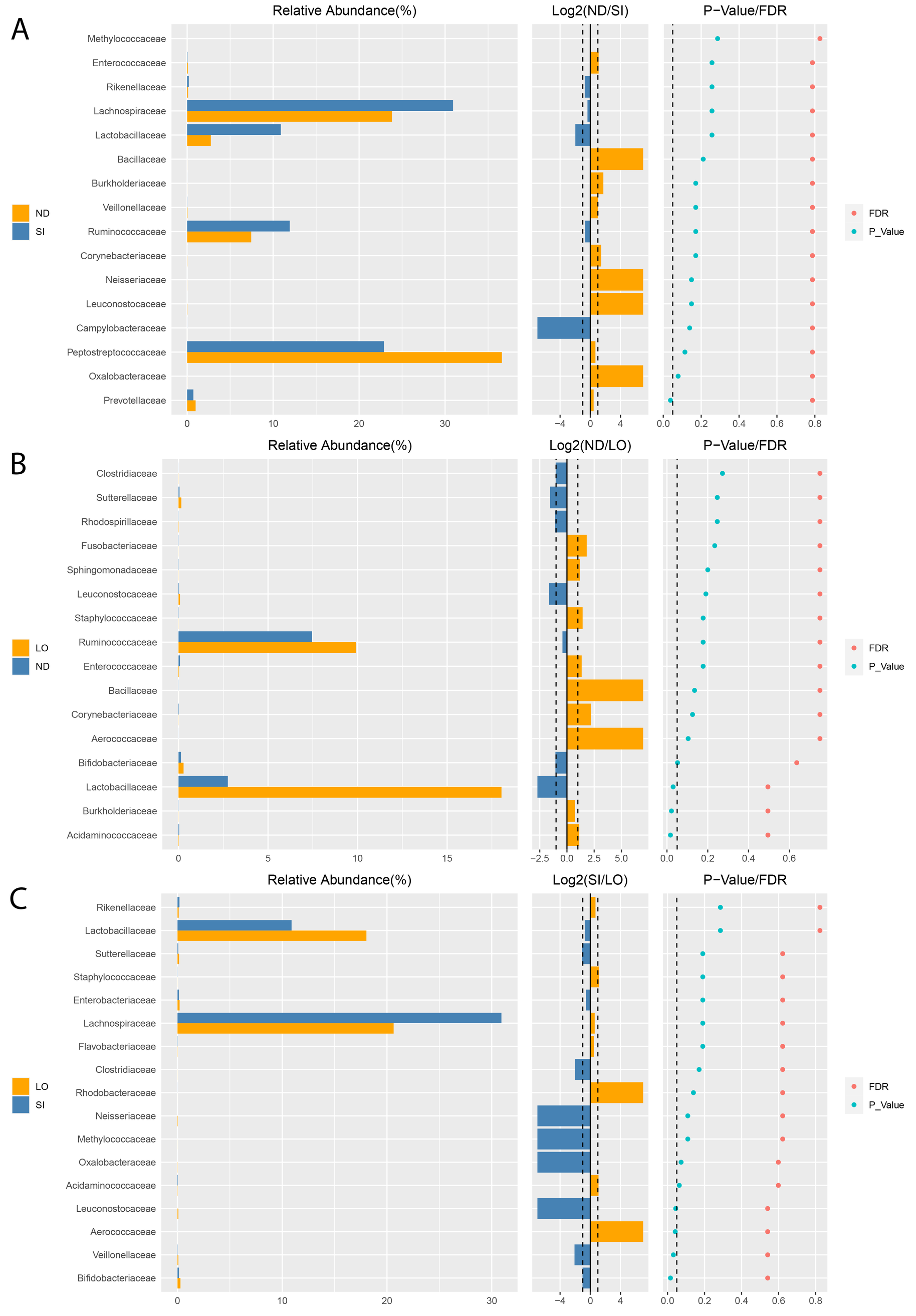

Supplement: S2 Fig — The difference in microbial relative abundance between the ND and SI groups (A), the ND and LO groups (B), and the SI and LO groups (C). (TIF) [file pone.0319066.s002.tif]

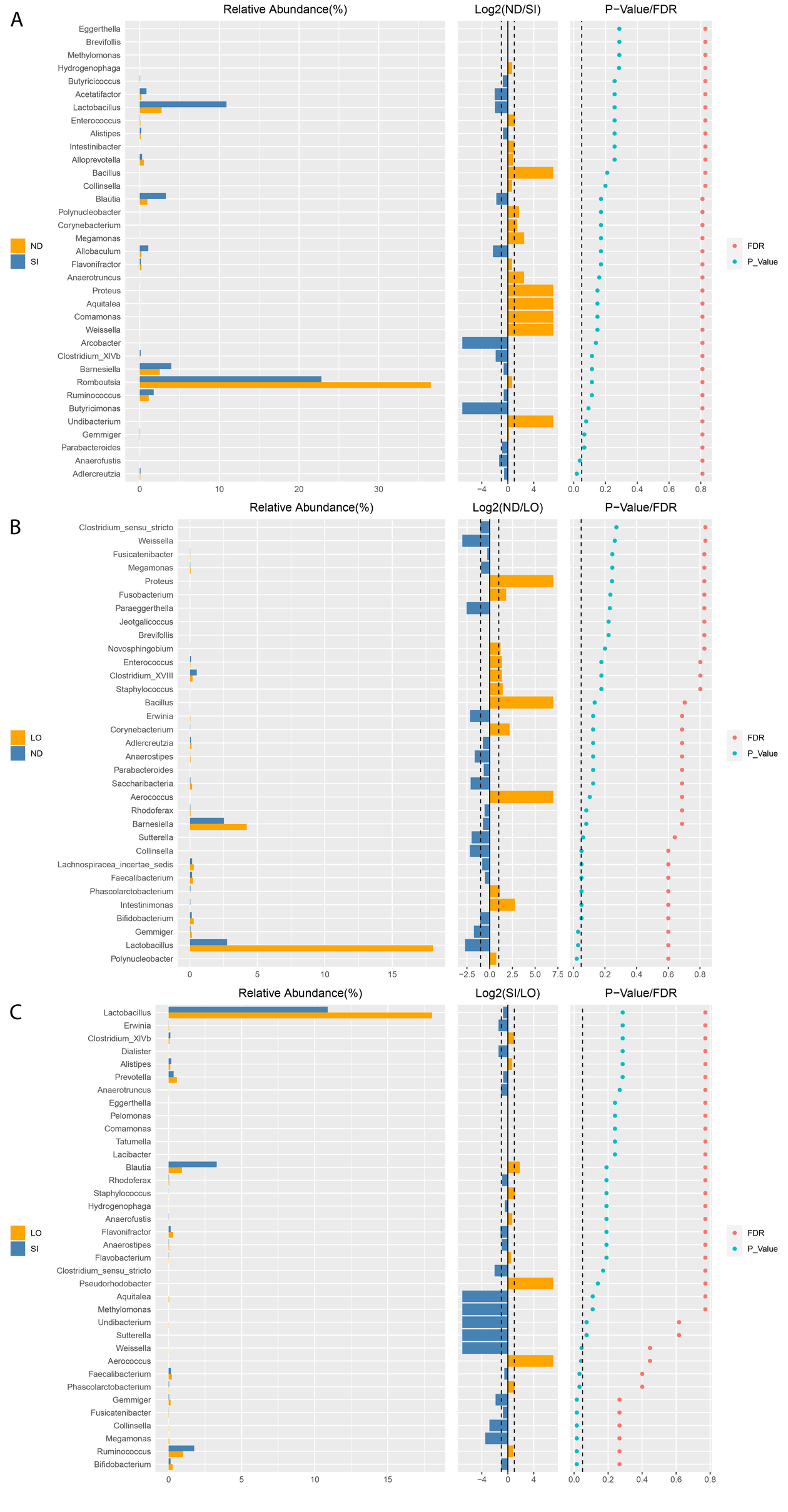

Supplement: S3 Fig — The difference in microbial relative abundance between the ND and SI groups (A), the ND and LO groups (B), and the SI and LO groups (C). (TIF) [file pone.0319066.s003.tif]

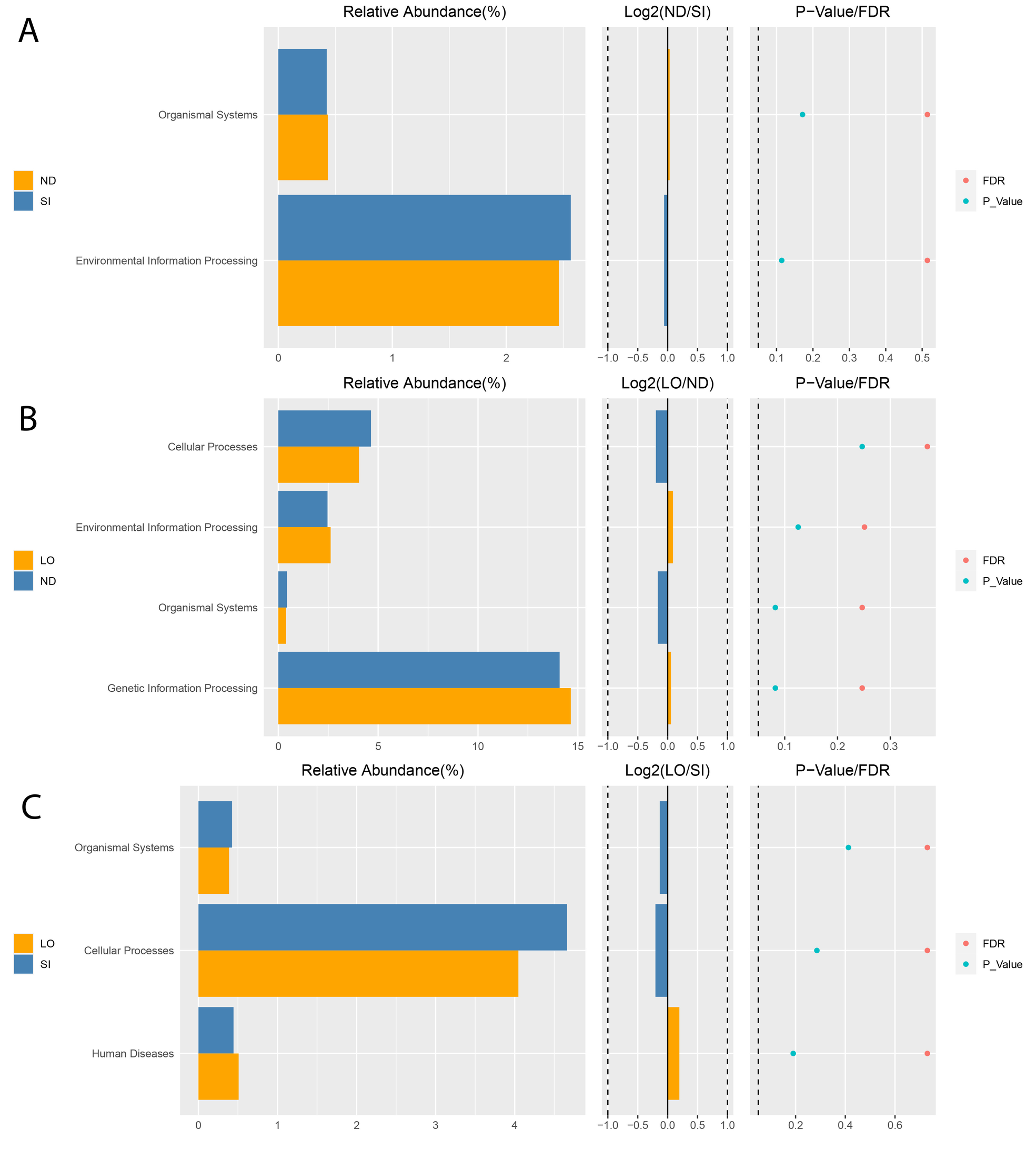

Supplement: S4 Fig — The bar plot presents the relative abundance of species. In the middle, log2 values represent the ratio of the average relative abundance between two groups. On the right, P-values and FDR values derived from the Wilcoxon test are displayed. Comparisons are made between ND and SI (A), LO and ND (B), and SI and LO (C). (TIF) [file pone.0319066.s004.tif]

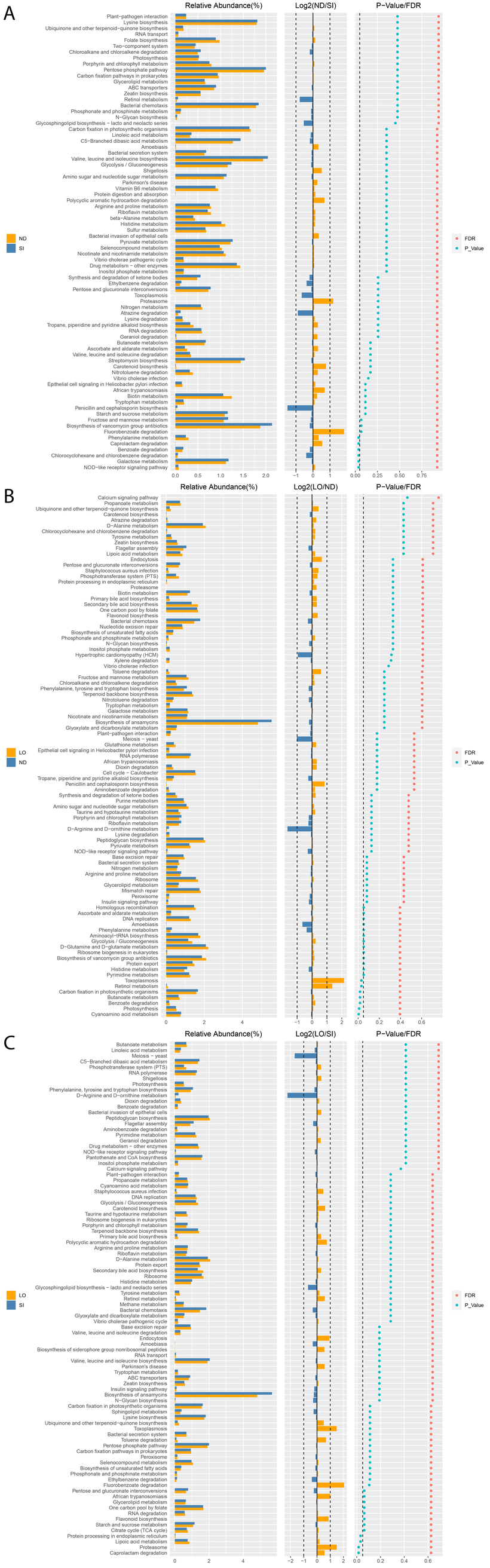

Supplement: S5 Fig — The bar plot presents the relative abundance of species. In the middle, log2 values represent the ratio of the average relative abundance between two groups. On the right, P-values and FDR values derived from the Wilcoxon test are displayed. Comparisons are made between ND and SI (A), LO and ND (B), and SI and LO (C). (TIF) [file pone.0319066.s005.tif]
