# Supplementary material for: Microbial effects of cold-pressed Sacha inchi oil supplementation in rats
Source: PLoS One. 2025 Feb 20;20(2):e0319066. doi: 10.1371/journal.pone.0319066 (PMC11841868; doi:10.1371/journal.pone.0319066)
Supplement: S1 Table — (DOCX) [file pone.0319066.s006.docx]

**S1 Table.** **The Naini and Cortina score of colonics in ND, SI and LO groups.**

| Features | ND group | SI group | LO group |
| --- | --- | --- | --- |
| Crypt architectural distortion | 0 | 0 | 0 |
| Basal lymph plasmacytosis | 0 | 0 | 0 |
| Cryptitis and crypt abscess | 0 | 0 | 0 |
| Increased lamina propria eosinophils | 0 | 0 | 0 |
| Erosions/ulcers | 0 | 0 | 0 |
| Granulomas | 0 | 0 | 0 |
| Paneth cell/pyloric metaplasia | 0 | 0 | 0 |
| Lymphoid nodules at base | 0 | 0 | 0 |
| Muscularis mucosae hyperplasia/splaying/disruption | 0 | 0 | 0 |
| Endocrine cell hyperplasia | 0 | 0 | 0 |
| Total colitis score | 0 | 0 | 0 |

The histomorphology of the colon in each group was determined by the histological activity score for inflammatory bowel disease, using the Naini and Cortina scoring system. This includes the grading of lesions in the colon (0 = absence; 1 = presence).
